# Dendritic Cells in Human Atherosclerosis: From Circulation to Atherosclerotic Plaques

**DOI:** 10.1155/2011/941396

**Published:** 2011-10-02

**Authors:** Emily A. Van Vré, Ilse Van Brussel, Johan M. Bosmans, Christiaan J. Vrints, Hidde Bult

**Affiliations:** ^1^Division of Cardiology, Department of Translational Pathophysiological Research, Faculty of Medicine, University of Antwerp, 2610 Wilrijk, Belgium; ^2^Division of Pharmacology, Department of Translational Pathophysiological Research, Faculty of Medicine, University of Antwerp, 2610 Wilrijk, Belgium

## Abstract

*Background*. Atherosclerosis is a chronic inflammatory disease with atherosclerotic plaques containing inflammatory infiltrates predominantly consisting of monocytes/macrophages and activated T cells. More recent is the implication of dendritic cells (DCs) in the disease. Since DCs were demonstrated in human arteries in 1995, numerous studies in humans suggest a role for these professional antigen-presenting cells in atherosclerosis. *Aim*. This paper focuses on the observations made in blood and arteries of patients with atherosclerosis. In principal, flow cytometric analyses show that circulating myeloid (m) and plasmacytoid (p) DCs are diminished in coronary artery disease, while immunohistochemical studies describe increased intimal DC counts with evolving plaque stages. Moreover, mDCs and pDCs appear to behave differently in atherosclerosis. Yet, the origin of plaque DCs and their relationship with blood DCs are unknown. Therefore, several explanations for the observed changes are postulated. In addition, the technical challenges and discrepancies in the research field are discussed. *Future*. Future studies in humans, in combination with experimental animal studies will unravel mechanisms leading to altered blood and plaque DCs in atherosclerosis. As DCs are crucial for inducing but also dampening immune responses, understanding their life cycle, trafficking and function in atherosclerosis will determine potential use of DCs in antiatherogenic therapies.

## 1. Introduction

Atherosclerosis takes a huge toll on our society. It is the leading cause of morbidity and mortality in the Western world, and growing incidence of atherosclerosis-related diseases has also been recently observed in developing countries [1]. It has become evident that inflammation mediated both by innate and adaptive immunity plays an important role even in the earliest stages of the development of atherosclerotic lesions [2, 3]. 

Dendritic cells (DCs) constitute a family of professional antigen-presenting cells that have the unique ability to induce primary T-cell responses. Moreover, they are not only essential in launching immune reactions against harmful antigens, but also in maintaining immune tolerance [4, 5]. As key modulators of immune responses, they are likely to play a crucial role in directing innate or adaptive immunity against altered self-antigens present in atherosclerosis, such as oxidized epitopes on apoptotic cells, oxidized low-density lipoproteins (oxLDL), or heat shock proteins (Hsp) [6]. 

**Figure 1:**
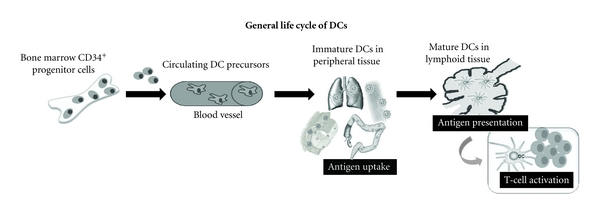
Schematic presentation of life cycle of DCs.

As illustrated in Figure 1, the general concept on the life cycle of DCs involves three stages. They originate from haematopoietic stem cells in the bone marrow and circulate as *precursors* in the blood stream, taking residence in target tissues at sites of potential antigen entry. Within these tissues, they give rise to *immature interstitial DCs* that act as sentinels, which continuously and efficiently sample the antigenic content of their microenvironment. In the steady state, immature DCs capture harmless self-antigens in the absence of inflammatory signals. They might enter the regional lymph nodes to present the self-antigen to naïve or resting T cells, which will be deleted by the induction of apoptosis, silenced by the induction of anergy or primed to become regulatory T cells [4]. In contrast, when infection and tissue damage occurs, immature DCs take up antigens in the presence of inflammatory signals, which cause activation and functional transformation into mature DCs, thereby downregulating endocytotic capacity and upregulating chemokine receptors (e.g., CCR-7), adhesion (e.g., CD50) and activation molecules needed for antigen presentation (e.g., CD83, CD86) [4, 5]. Meanwhile they exit the nonlymphoid tissues to migrate via afferent lymph to lymphoid tissues (lymph nodes or spleen), where they complete maturation. *Mature DCs* will present short peptide fragments, which are bound to the surface molecules CD1 or major histocompatibility complex- (MHC-) I or MHC-II. Consequently, they will activate (naïve) T and B lymphocytes that recognize the presented antigen [5, 7–9]. 

The present review is aimed at summarizing current knowledge of the role of DCs in the pathogenesis of human atherosclerosis: from circulating DC precursors in patients with coronary artery disease (CAD) to DCs found in human atherosclerotic lesions. Technical challenges and open questions in this research field are discussed in detail.

## 2. Circulating DCs in CAD

### 2.1. Subtypes of Blood DC Precursors

Two main DC precursor subtypes can be identified in human blood: myeloid (m)DCs and plasmacytoid (p)DCs. As DC precursors they are relatively immature and express only low levels of adhesion and costimulatory molecules—at least in physiological conditions [10–12]. mDCs (0.26% among leukocytes) descend from the myeloid lineage, and express blood DC antigen (BDCA)-1 (= CD1c), CD11c, and Toll-like receptors TLR2, TLR4, TLR5, and TLR3 [13–15]. They secrete mainly IL-12 in response to bacterial components such as peptidoglycans, lipopolysaccharide (LPS) or flagellin, and extracellular bacterial DNA, respectively. Unlike mDCs, pDCs (0.2% among leukocytes) express BDCA-2 (= CD303) and CD123, and are specialized in innate antiviral immune responses by producing copious amounts of type I interferons upon exposure of intracellular TLR9 and TLR7 to DNA and RNA viruses, respectively [16–18]. Apart from responding to different pathogen-associated molecular patterns (PAMPs) and secreting different cytokines, mDCs and pDCs also differ in migration behaviour [16, 17, 19–22]. Generally it is assumed that mDCs are the conventional DCs that infiltrate peripheral tissues while pDCs migrate directly from the blood into lymphoid organs. 

Finally, a small (0.02% of leukocytes) third population of blood DCs expressing CD11c, and BDCA-3 (= CD141) but not BDCA-1, CD123 and BDCA-2 can be distinguished [23]. BDCA-3^+^ mDCs are far less studied than the classical BDCA-1^+^ mDCs, but recent reports stress their unique function and importance. They emerge as a distinctive myeloid DC subset that is characterized by high expression of TLR3, production of IL-12 and IFN-*β*, and a superior capacity to induce T helper-1 cell responses, when compared with BDCA-1^+^ mDCs [24]. Moreover, BDCA-3^+^ mDCs are endowed with the capacity to cross-present necrotic cell antigens and induce cytotoxic T-cell responses [24].

### 2.2. Decline of Blood DCs in CAD

In 2006 we reported for the first time a decrease in circulating DC precursors (BDCA-1^+^ mDCs, BDCA-2^+^ pDCs) in patients with coronary artery disease (CAD), the clinical manifestation of atherosclerosis [25]. CAD was determined by angiography and defined as more than 50% stenosis in one or more coronary arteries. Blood DCs were enumerated by multicolour flow cytometry. Absolute and relative numbers of circulating BDCA-2^+^ pDCs were significantly lower (55% decline) in 18 CAD patients, compared to 18 age- and sex-matched healthy volunteers. Absolute BDCA-1^+^ mDC numbers tended to be smaller in patients, while relative numbers were significantly diminished (21% decline). Simultaneously and independently, Yilmaz et al. [26] also found a marked reduction in mDC precursors in CAD patients, though the decline in pDCs was less pronounced. These minor differences between both early reports may be due to insufficient power of those studies. In addition, Yilmaz et al. showed that patients with acute myocardial infarction (AMI), which were excluded by van Vré et al. [25], had the most dramatic decline (63%) in blood mDCs [26]. However, this may be in response to development of myocardial necrosis rather than atherosclerosis in se. Moreover, it may be due to the doubling of the total leukocyte count after AMI, resulting in lower relative numbers of mDCs in patients with AMI, but comparable absolute counts between AMI patients and patients with stable CAD. This illustrates an important technical weakness of flow cytometric enumeration and stresses the importance of fairly stable unaltered total leukocyte counts, as flow cytometry determines the relative fraction of DCs within the total leukocyte population. Otherwise it would be preferable to add fluorescent beads as internal standards to the blood sample, which allows for absolute cell counting.

Subsequently, we investigated whether the decline of blood DCs in CAD patients was related to the number of diseased vessels (one- versus three-vessel disease) or type (stable versus unstable angina pectoris) of CAD [27]. This study also determined total blood DCs, identified as lineage (CD3, CD14, CD16, CD19, CD20, CD56) negative and HLA-DR positive. Again relative and absolute numbers of pDCs and mDCs were significantly lower (35 and 34%, resp.) in patients with coronary atherosclerosis. Interestingly, the overall lineage^−^ HLA-DR^+^ blood DCs, which also include other blood DCs (e.g., BDCA-3^+^) or more mature blood DCs, confirmed the decline of BDCA^+^ DC precursors. However, the counts of circulating DCs dropped to the same extent in three groups of CAD patients, irrespective of the number (one or three) of affected arteries or the type (stable or unstable) of angina. In analogy, Yilmaz et al. found no differences between clinically stable or unstable CAD [26]. Yet, in a subsequent more extended study [28] with a cohort of 290 patients, they found that the numbers of pDCs, mDCs, and total DCs declined as the extent of coronary atherosclerosis increased. In that study they examined patients with suspected stable CAD, but used a more refined “CAD score.” They assessed the maximum grade of stenosis in 15 well-defined segments of the three coronary arteries and their main side branches. These values were counted up to obtain an overall CAD score, and then patients were subdivided into 4 groups: CAD excluded, early CAD, moderate CAD, and advanced CAD. Moreover, they showed that the decrease in DC numbers was an independent predictor of the presence of CAD when several risk factors (age, male gender, diabetes, and hypertension) were included [28]. 

Surprisingly, Shi et al. [29] using CD11c and CD123 rather than BDCA-1 and BDCA-2 as blood DC markers, described increased CD11c^+^ and unchanged CD123^+^ DC numbers in men with stable CAD. Yet, recently we underlined that circulating DCs decline in CAD, irrespective of the subset marker (BDCA-1 or CD11c for mDCs, BDCA-2 or CD123 for pDCs) that was used for enumeration [30]. No details on timing of blood sampling are mentioned by Shi et al. It may be that in the CAD patients blood samples were taken immediately after a percutaneous coronary intervention (PCI). Since we noticed that PCI resulted in decreased leukocyte numbers shortly after the intervention (unpublished data, Van Vré et al.), this may have invoked a rise of the relative proportions of DC counts. Therefore, with respect to standardisation and to minimize interfering factors, blood sampling before the intervention seems preferable.

### 2.3. Possible Explanations for the Decline of Blood DCs in CAD

**Figure 2:**
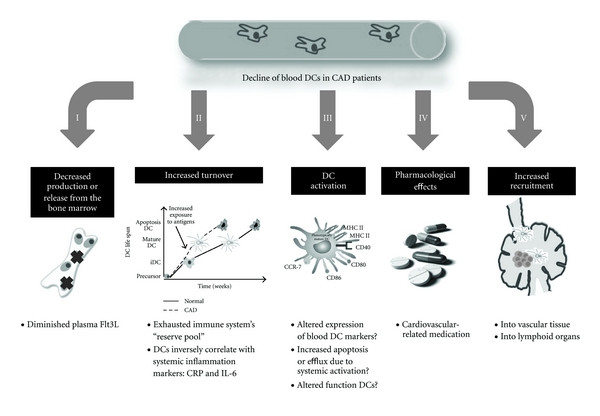
Possible mechanisms responsible for the decline of blood DCs in atherosclerosis.

At the moment the mechanisms responsible for the decline of blood DCs in atherosclerosis are still unclear. Different possibilities are discussed below and summarized in Figure 2.

#### 2.3.1. Impaired Differentiation from Bone Marrow Progenitors

Decreased production or release from the bone marrow could result in reduced blood DC precursor numbers. As discussed above, DCs are also necessary for induction of tolerance against harmless antigens [5, 31]. Consequently, a diminution in DC precursors could promote the development of (auto)immune disease, such as atherosclerosis [32]. Interestingly, we recently showed diminished plasma Flt3 ligand (Flt3L) concentrations in CAD [12]. Flt3L is a major cytokine involved in both pDC and mDC development from haematopoietic stem cells and their release from the bone marrow [33–35]. As plasma Flt3L correlated with blood DC counts, the reduced blood DCs in CAD might be caused by impaired DC differentiation from bone marrow progenitors. Until now, it remains unclear why plasma Flt3L levels are lowered in CAD. In contrast, plasma concentrations of the DC growth factor GM-CSF were similar in CAD patients and controls and did not correlate with mDC or pDC counts [12], indicating that it is unlikely that GM-CSF accounts for the decreased numbers of blood DCs in human CAD. 

#### 2.3.2. Increased DC Turnover

Another explanation for the decreased DC numbers could be the result of increased turnover, that is, decreased survival or production versus apoptosis rates. Atherosclerosis is a chronic disease, evolving over several decades with inflammatory reactions taking place from the earlier stages. It is plausible that in the end as symptoms emerge and the exposure to (new) antigens—derived from stressed and dying cells, lipid, or protein modifications due to oxidative stress in the plaque—increases, the immune system's “reserve pool” has become exhausted. For instance it was demonstrated that oxidized low-density lipoprotein, one of the main antigens present in plaques and in the circulation of atherosclerotic patients, may cause increased apoptosis of DCs [36]. Interestingly, no decline was observed [25–27] for other blood cells, such as monocytes, pointing to a specific role of DCs in atherogenesis. As DCs form the major patrol system of the body, involved in first-line defence, innate and adaptive immunity, they may be the most sensitive/vulnerable to local and systemic changes, or neoepitopes, presented during atherosclerosis. In the end, this may result in a system that is no longer able to sustain the number of blood DCs. Indeed, numbers of circulating blood DCs, in particular pDCs, decline with increasing age (unpublished data, Van Vré et al.). However, when age was included as covariate, the decline of mDCs and pDCs in CAD patients appeared to be independent of the effect of age [27].

#### 2.3.3. DC Activation Leading to Loss of Subset Markers

Activation of blood DCs by factors in the circulation (e.g., oxLDL) could account for diminished blood DC numbers by reducing the expression of precursor markers. Indeed, it has been described that pDC maturation results in complete BDCA-2 downregulation [23, 30, 37], and this could potentially lead to underestimation of circulating pDC numbers. In addition, we recently showed that *ex vivo* DC activation resulted in an increase in CD11c on mDCs and CD123 on pDCs [30]. Thus, when different DC numbers are found between study populations, the DC activation status needs to be verified, since the numerical changes may result from the altered expression of the subset markers during activation [30]. Yet, by using CD11c/BDCA-1 and CD123/BDCA-2 ratios to assess DC activation, no differences were found between controls and CAD patients [30]. From this it was concluded that there was no indication for overt activation of DC precursors in patients with CAD.

#### 2.3.4. DC Activation Leading to Increased Extravasation

Though several studies investigated numbers of subsets of DCs in the circulation of CAD patients, very little additional information is available on the status of maturation and activation in circulating DCs. It is possible that in inflammatory conditions systemic activation occurs in the blood and this could lead to increased extravasation or apoptosis of blood DCs. Interestingly, inverse associations of circulating mDCs, pDCs (and total DCs) were found with blood markers of inflammation: CRP and IL-6 [25–28]. Upon stimulation by circulating oxLDL or other atherosclerosis-related modified proteins, blood DCs may become activated, upregulate chemokine receptors such as CCR-7, and then travel towards lymphoid organs or inflamed tissues, such as atherosclerotic plaques. 

Yilmaz et al. assessed the activation status of blood DC precursors and reported a weak expression of costimulatory molecules CD40 and CD86 on circulating BDCA-1^+^ mDCs or BDCA-2^+^ pDCs [26], without differences between control and CAD patients. We also detected very few activated blood DCs: a minority of circulating mDCs (14–22%) and pDCs (14–20%) had a more mature phenotype and expressed but low levels (MFI) of CD83, CD86, and/or CCR7. Nevertheless, we showed that the frequency of CD86 and CCR-7 expressing mDCs was less in CAD patients whereas this was not seen in pDCs [12]. This could point to increased efflux or apoptosis of activated blood CD86^+^CCR-7^+^ mDCs, contributing to their decline in blood.

#### 2.3.5. Functional Capability of Circulating DC Precursors in CAD

As blood DC precursors from CAD patients remained fairly immature, we investigated their capability to achieve maturation. Therefore, we incubated whole blood samples with TLR ligands *ex vivo*, and then evaluated expression of activation markers by means of flow cytometry. Stimulation with the TLR-4 ligand LPS upregulated the expression of CD83 and CD86 on mDCs, without changing CCR-7 expression or IL-12 secretion. Moreover, there were no differences between mDCs from CAD patients or controls. For pDCs, patients showed a weaker upregulation of CD83 and less IFN-alpha secretion upon stimulation with imiquimod, a TLR7 ligand. Hence, these results indicate that although mDCs decline in CAD patients, they appear to be functioning in a normal way. In contrast, pDCs from CAD patients are not only reduced in numbers, but also seem subactive [12]. 

Finally, as monocytes can serve as precursors for DCs in peripheral tissues [38], three groups used a completely different approach and studied the function of monocyte-derived (mo)DCs in CAD. They isolated peripheral blood monocytes from CAD patients and differentiated them ex vivo for several days with GM-CSF and IL-4. moDCs from patients with coronary syndromes, particularly unstable angina, appeared to be more activated compared to moDCs from controls or healthy volunteers [39–41]. These data suggest that monocytes from CAD patients when exposed to DC growth factors in atherosclerotic lesions will be more prone to differentiate into activated DCs.

#### 2.3.6. Drug-Induced Changes in Blood DCs

Cardiovascular disease-related medication, taken by CAD patients, might influence numbers, phenotype, or function of blood DCs in atherosclerosis. Indeed, several *in vitro* studies show potential effects of cardiovascular drugs on DC maturation and function.

*Aspirin* promotes early moDC differentiation to functionally active, immature DCs, and strongly maintains these cells in an immature state [42–44]. *In vivo* experiments in mice demonstrated an impairment of cell-mediated immune responses, which was not due to the inability of aspirin-treated DCs to migrate to drained lymphoid tissue [42]. In contrast to immature DCs, fully differentiated DCs were not susceptible to inhibition by aspirin [43]. The latter indicates that it is the process of differentiation, rather than the function of mature DCs, which is the target of aspirin. 

Kofler et al. demonstrated that *statins* inhibited adhesion and transmigration of moDCs through dysfunctional endothelial cells *in vitro* [45], suggesting that under statin treatment less DCs would accumulate in atherosclerotic plaques. Although statins have been reported to suppress DC maturation and function, [46–48], data on *in vitro* DC activation in the presence of statins remain rather conflicting. It seems that statins partially suppress *in vitro* DC maturation by inhibition of the NF*κ*B activity [48], but may affect DC function differently [46] depending on the maturation parameters analysed and the type of statin tested. 

Studies on the effects of *beta blockers* on DC maturation and function are absent, though beta blockers may reverse changes in leukocyte distribution. Von Haehling et al. demonstrated that the decrease in lymphocytes and the increase in neutrophils in patients with chronic heart failure are less pronounced when patients are on a beta-blocker therapy [49]. After distal type acute aortic dissection, early use of beta blockers prevented excessive inflammation, indicated by lower maximum white blood cell counts and lower serum CRP levels. This suggests pleiotropic effects of beta blockers on the inflammatory response [50]. 

Also angiotensin-converting enzyme- *(ACE-) inhibitors* and *calcium entry blockers*, two groups of pharmaceuticals that are used primarily in treatment of hypertension, might influence DC function. ACE-inhibitors were shown to suppress LPS-induced proinflammatory cytokine secretion of moDCs [51] whereas calcium entry blockers prevent apoptotic body engulfment by DCs and inhibit IL-12 secretion [52, 53]. Yet, calcium entry blockers did not affect the capacity of antigen-presenting cells to prime naive T cells or to induce T helper-2 cell proliferation, nor the capacity of antigen-presentation by DCs [54]. Hence, both classes of anti-hypertensive drugs may suppress DC function, but are less commonly used in the treatment of CAD patients.

If these *in vitro* studies on the effects of cardiovascular drugs on human moDCs and mouse bone-marrow-derived DCs can be translated to CAD patients, they would affect blood DC numbers. As aspirin stimulates early differentiation of CD11c^+^ DCs from bone marrow progenitors, aspirin therapy might lead to increased circulating DC numbers rather than decreased numbers. Furthermore, if statins inhibit the adhesion and transmigration of DCs through activated endothelial cells, this would result in less recruitment of DCs in atherosclerotic plaques, and thus retention of DCs in the circulation. Indeed, we showed recently that use of statins was associated with increased numbers of mDCs whereas pDC counts were unaffected [27]. Also DC activation is to some extent inhibited by statins, aspirin, ACE-I, and calcium entry blockers, which might explain their immature phenotype in an inflammatory condition as atherosclerosis. Finally, as beta blockers might reverse changes in leukocyte distribution and have pleiotropic effects on inflammatory responses, the decline of circulating DCs in CAD may be less pronounced in patients on a beta-blocker therapy as well.

In a recent study, we analyzed the effect of medication by including “control patients,” that is, patients with chest pain and suspected CAD, who appeared to have coronary arteries with less than 50% stenosis, instead of healthy volunteers [27]. This control population did not significantly differ from CAD patients with respect to the intake of medication. Yet, patients with proven CAD had lower blood counts of both subsets of DCs, strengthening the idea that their decline in CAD is not a bystander effect of cardiovascular-related drugs. Yilmaz et al. came to the same conclusion for statins, aspirin and beta blockers [28]. Moreover, in a factorial analysis of variance lipid-lowering drugs and beta blockers augmented, rather than decreased, the number of circulating mDCs in CAD, without affecting pDCs. Aspirin, angiotensin-converting enzyme ACE/AII inhibitors and calcium entry blockers had no impact on DC numbers [27]. As this was only a limited pilot study, more studies are required to investigate the impact of medication on circulating DC numbers and function.

#### 2.3.7. Increased Recruitment of DCs from the Circulation to Inflammatory Sites

An attractive explanation for decreased circulating DC numbers might be increased recruitment of DCs into the vessel wall or lymphoid organs. Indeed it has been mentioned that DC numbers of lymph nodes attached to atherosclerotic wall segments exceed those in lymph nodes attached to nonatherosclerotic arteries [7]. As to the presence of DCs in healthy and diseased arteries, several studies have been done and these are discussed in the second part of this review.

## 3. DCs in Atherosclerotic Lesions

**Figure 3:**
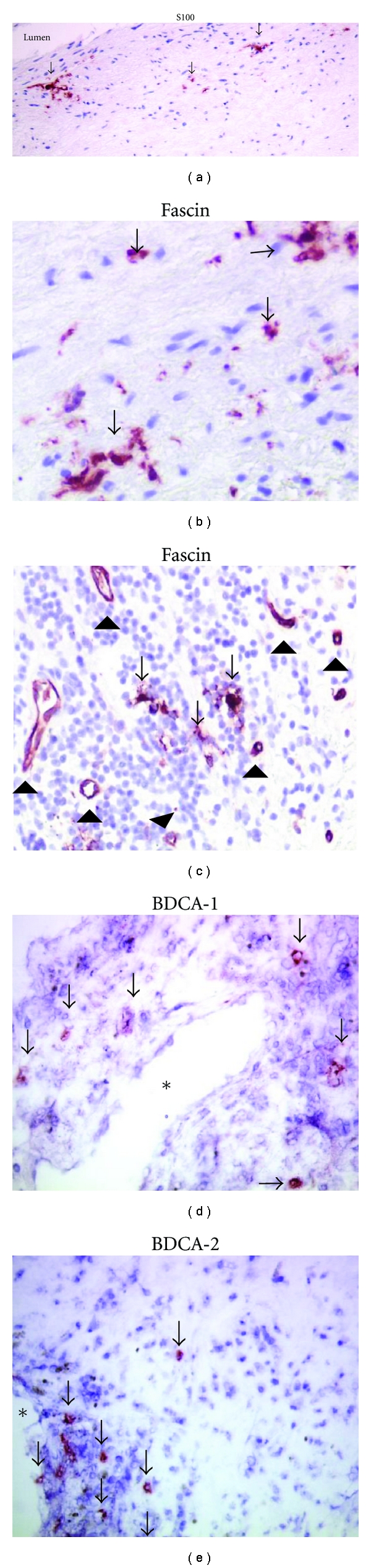
Examples of useful plaque DC markers: S100 (a), fascin (b, c), BDCA-1 (d) and BDCA-2 (e). Arrows indicate DCs. Arrowheads show fascin^+^ neovessels. ∗ indicates lumen of microvessel.

The presence of DCs in human arteries was first described by Bobryshev and Lord 1995 [55–57]. By electron microscopy they demonstrated distinct morphologic characteristics that allow unambiguous identification of “vascular DCs” [7, 55, 58, 59]. In addition, DCs in human arteries were identified using immunohistochemical staining for S100 (Figure 3(a)) [56], langerin [60], CD1a [61], and fascin (Figure 3(b)) [62], which identify immature/mature subsets of DCs. More recent histological studies added a general (immature and mature) DC marker: DC-SIGN [63, 64] and mature DC markers, such as DC-LAMP [65], CD1a [58, 60], and CD83 [65–67]. Therefore, as suggested above, an attractive explanation for the decline in blood DCs is active recruitment into atherosclerotic lesions. Indeed, the presence of blood DC markers BDCA-1, BDCA-2 [26, 68, 69], CD11c, and CD123 [70–73] has been described in atherosclerotic plaques. Nevertheless, as summarized in Table 1 and discussed below, there remains a lot of uncertainty on quality and specificity of immunohistochemical DC markers.

### 3.1. Pitfalls When Examining DCs in Atherosclerotic Plaques

At first, it became clear that *autolysis* in autopsy specimens and *fixation* destroy or shield most cell surface (CD) epitopes [69]. This means that nearly all DC markers cannot be studied in formaldehyde-fixed human autopsy specimens, and therefore information on their presence in early plaque stages is lacking. For these surface markers (e.g., CD83, and BDCA-1, BDCA-2) only freshly frozen, unfixed endarterectomy specimens yield reliable results [69].

Secondly, false-positive results, resulting from *unspecific binding of antibodies to plaque components *often occur in atherosclerotic plaques [82]. The oxidative processes in advanced plaques lead to creation of hot spots of multiple neoantigens, and these may bind primary or secondary antibodies, particularly if used in inappropriate dilutions. Therefore, optimized immunohistochemistry protocols are an absolute prerequisite. For instance CD83 [63, 67, 69] and BDCA antigens [69] could be detected in human plaques [69], but they were less eminently present than reported by others [26, 65, 83]. Actually we suspect that in some studies the primary antibody against those epitopes was far too concentrated, and even combined with signal amplification methods that will increase background staining even further. 

Thirdly* the lack of DC specific markers* makes identification of vascular DCs by means of immunohistochemistry far more difficult than with flow cytometry in which a panel of markers enables unequivocal positive or negative (for other lineages) detection of various cell types. For instance, the markers CD11c and CD123, which have been used to detect blood DCs in plaques [70, 84], are also expressed by, respectively, monocytes/macrophages and endothelial cells [69]. Fascin is most frequently used to detect vascular DCs [62, 65, 66, 68, 73–76], but we recently showed abundant fascin expression in endothelial cells and neovessels in plaque shoulders and in complicated lesions [69, 77] (Figure 3(c)). Also the lipid antigen presenting molecule, CD1a appeared to be unspecific for plaque DCs as it identified also foam cells, CD14^+^  monocytes, and CD68^+^ macrophages [69, 81], whereas the activation marker CD83 can be present on T-cells or activated monocytes and macrophages in advanced plaques [69, 85, 86]. Though double immunofluorescence staining can be used to check multiple markers on individual cells, the very high levels of autofluorescence in atherosclerotic plaques make routine application of this technique impossible. At the moment we propose S100 as the most reliable and general marker of DCs in plaques DCs (Figure 3(a)), but not in the adventitia where it stains nerve twigs. Fascin may yield reliable results (Figure 3(b)), but only in early lesions that are still devoid of intraplaque angiogenesis.

Finally, there is *no universal DC marker *that is expressed by all DCs. Indeed, few DCs simultaneously expressed the most reliable markers fascin and S100 [69], and the presence of DCs as detected by immunohistochemistry is markedly lower than DC numbers identified by electron-microscopy [55, 58]. Therefore, one has to keep in mind that quantification of intimal DCs by one marker will only give information on a particular DC subtype.

### 3.2. Different DC Subtypes in Atherosclerotic Lesions

#### 3.2.1. DC Precursors in Advanced Atherosclerotic Plaques

Interestingly, both mDC and pDC precursor markers were found in carotid atherosclerotic plaques, although less BDCA-2^+^ (pDC marker) cells were present [69]. Yilmaz et al. described comparable results in atherosclerotic coronary arteries [26]. These findings are substantiated by the fact that gene expression analysis for pathogen-sensing Toll-like receptors (TLRs) 1 to 9 showed vessel-specific profiles, with the mDC receptors TLR2 and TLR4 ubiquitously present, but the pDC receptors TLR7 and TLR9 infrequently so [87]. Niessner et al. described the presence of plaque residing CD123^+^ pDCs in advanced plaques, suggesting that they can amplify cytolytic T-cell functions and may thus connect host infection and plaque instability [70].

The demonstration of BDCA-2^+^ and BDCA-1^+^ cells in human plaques strongly suggests that blood DCs are recruited into advanced lesions. This is further strengthened by the observation that both BDCA^+^ subsets were predominantly found around microvessels [69]. At the moment there still is a lack of information on their potential presence in early atherosclerotic lesions, due to the fact that their markers cannot be investigated in fixed arteries.

#### 3.2.2. Immature/Mature DCs in Atherosclerotic Plaques

S100^+^ and fascin^+^ DCs are found in normal arteries and in all successive stages of atherosclerotic lesions [7, 59, 65, 69]. Interestingly, atheroprone regions (subjected to hemodynamic stress) contain more DCs than atheroresistant areas [88]. Small numbers of DCs are located along the endothelium in the subendothelial layer of the intima of apparently normal, nondiseased arteries, even in children [78, 89]. Wick et al. proposed that these intimal DCs are part of a “vascular associated lymphoid tissue” (VALT), which also consists of T cells and macrophages, and screens the vessel wall for potentially harmful antigens [32, 90, 91]. 

Though the presence of S100 in normal intimal thickenings is limited, numbers of S100^+^ cells increase successively from intimal thickening, via pathological intimal thickening, fibrous cap atheroma and finally complicated plaques [56, 69]. Fascin^+^ cells follow the same pattern, but are more abundant [26, 69, 76, 83]. For early plaque stages this is a real finding, but in lesions containing microvessels (plaque shoulders, complicated plaques, and most endarterectomy specimens) this was partly explained by staining of fascin-positive endothelial cells [69].

#### 3.2.3. Mature, Antigen-Presenting DCs in Atherogenic Lesions

The report on abundant presence of CD83^+^ cells in plaque shoulders [65] is often cited as evidence that atherosclerotic plaques contain many activated DCs. However the markers CD1a and CD83 are technically difficult or less specific for detection of mature DCs. Therefore, it is in our view still debatable whether mature DC counts augment when plaque stages increase. Nevertheless, mature DCs can be found in advanced lesions, and it became clear that DCs form close interactions with T cells in advanced atherosclerotic lesions, suggesting that *in situ* T-cell activation does occur [65, 67, 69, 74].

### 3.3. Possible Mechanisms for Increased DCs in Atherosclerotic Arteries

**Figure 4:**
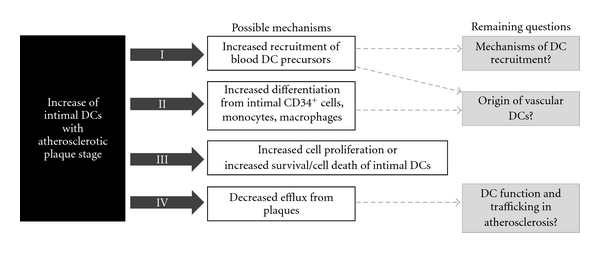
Possible mechanisms responsible for the increase of vascular DCs in atherosclerosis.

Different explanations for increased DC numbers in atherosclerotic lesions are discussed below and summarized in Figure 4. 

#### 3.3.1. Increased Influx of Blood DC Precursors

At first and in view of the many reports on diminished blood DCs in patients with established atherosclerosis, one likely explanation is increased invasion of DC precursors from the blood into the arterial lesions. This hypothesis is strengthened by the present markers of pDCs and mDCs around neovessels. Future studies will have to determine whether a link exists not only between plaque type and numbers of lesional DCs, but also with blood DC counts. It is possible that, the clinical division in stable or unstable, was not refined enough to detect this correlation. Ideally, DCs have to be investigated in peripheral blood and plaque samples of the same patient, or in an animal model of experimental atherosclerosis, with fluorescence labeling of DCs, to characterize their origin, to postulate an increased influx of blood DC precursors as a mechanism for increased DC numbers in atherosclerotic plaques. Direct information on the stimuli regulating DC migration is scarce. However, it is known that effective mDC as well as pDC chemokine factors (CCL2, CCL5, and CXCL-12) are elevated in patients with atherosclerosis [20–22, 92]. In addition, endothelial cell adhesion molecules such as E-selectin, P-selectin, and vascular cell adhesion molecule-1 are increased in atherosclerosis and can enhance invasion of monocytes or DC precursors [68]. Indeed, endothelium activated *in vitro* by atherogenic factors such as oxLDL or TNFalpha increases moDC adhesion and endothelial transmigration [93]. Furthermore, we recently showed that endothelial cell dysfunction in humans (measured by brachial vasodilatation in response to reactive hyperaemia) does have an impact on the number of circulating mDCs. Poor vasodilatation increased mDCs (but not pDCs) in the blood, on top of the reduction evoked by CAD [27]. Finally, experimental studies in mice suggest that DCs invade the plaque via the arterial lumen [94] while the proliferation rate of DCs in plaques is very low [95]. Interestingly, deficiency in CX3CR1—a receptor of the chemokine fractalkine—in mice impaired the accumulation of DCs in the aortic wall and this markedly reduced the atherosclerotic burden [96].

#### 3.3.2. Increased Differentiation from Intimal CD34^+^ Cells or Monocytes

In spite of these indications that blood DC precursors might account for increased plaque DC numbers, the origin of immature/mature (S100^+^ or fascin^+^) DCs remains unclear [7]. Until now it has not been shown that DC precursors can give rise to plaque DCs [38]. Therefore, a second and third explanation for the augmented presence of DCs in atherosclerotic plaques are increased differentiation of DCs from local vascular CD34^+^ stem cells [59] or from infiltrated monocytes [38, 59, 95, 97]. Interestingly, aortic lesions of GM-CSF-deficient low-density lipoprotein receptor null mice had a dramatic 60% decrease in CD11c^+^ DCs, suggesting that GM-CSF might be an important local factor that determines DC numbers in the plaques [98]. In addition, several studies already pointed to the fact that atherogenic factors such as oxLDL, phospholipase A2 type IIA, and CRP could interfere with DC differentiation from monocytes [99–101]. Moreover, next to monocytes also macrophages and foam cells can turn into DCs [99, 102]. As discussed below, this transition might be necessary to enable migration to lymph nodes and plaque regression [103, 104]. 

#### 3.3.3. Defective Emigration of DCs from Plaque to Lymphoid Organs

It was recently suggested that the accumulation of DCs in plaques could be the result of defective emigration of DCs from the lesions to draining lymph nodes [38, 105]. In a mouse model of dyslipidemia it was shown that altered serum lipids associated with atherosclerosis changed DC activation and migration [106]. Further evidence was given by transplantation of atherosclerotic segments from hypercholesterolemic ApoE^−/−^ mice into wild-type normocholesterolemic recipient mice. This resulted in lesion regression whereas lesion development continued progressively after transplantation into hypercholesterolemic mice [104, 107, 108]. The rapid loss of plaque volume after transplantation of segments into normocholesterolemic mice, was explained by emigration of DCs to lymph nodes [104, 109], while DC exit from aortic lesions was impaired in hypercholesterolemic mice [104]. Trogan et al. demonstrated that CCR7 was increased after transplantation of the plaques in normolipidemic mice [103]. In contrast, when CCR7 function was abrogated *in vivo* by treatment with antibodies to CCR7 ligands CCL19 and CCL21, lesion size and foam cell content did not regress [103]. Doherty et al. explained that—in accordance with giant cell arteritis [110]—trapped intimal DCs may not only trigger local inflammation, but it may also lead to loss of local tolerance [105]. Nevertheless, in human atherosclerotic arteries DCs can be regularly observed trafficking through the media and the internal elastic lamina [69, 74], but it is not known whether the rate of cell fluxes has changed.

## 4. General Conclusion

This review summarizes the current understanding of the possible role of DCs in the pathogenesis of human atherosclerosis. Concerning the early stages of DC differentiation, that is, circulating DC precursors, it is now unambiguous that they are significantly decreased in CAD patients, irrespective of the blood DC markers used for enumeration. Exact mechanisms responsible for their decline remain unclear. However, there are indications pointing to impaired differentiation from bone marrow progenitors, and to activation and subsequent recruitment to inflammatory sites, such as atherosclerotic plaques. Indeed, both mDCs and pDCs can be found in human plaques, particularly around neovessels in areas with angiogenesis. Furthermore, DC counts in the intima of arteries increase with evolving plaque stages, and activated DCs are seen in close relationship with lesional T cells. To which extent these interactions between DCs and T cells result in progression or dampening of atherosclerosis is, however, not yet clear. Interestingly, it has been demonstrated in mice that decreased accumulation of DCs in the arterial intima can lead to attenuated plaque progression [96]. However, a protective role for DCs in atherosclerosis by regulating cholesterol homeostasis [111] has also been shown. Furthermore, increasing evidence points to different behaviour of mDCs and pDCs in atherosclerosis. The mDCs from CAD patients and controls responded similar to *ex vivo* stimulation, while pDCs from CAD patients were subactive. To elucidate possible atheropromoting or protective functions of blood DCs in humans, studies investigating the relationships between plaque composition, size, and stability by means of IVUS, and numbers and functional status of circulating pDCs and mDCs would be helpful. However, in the end experimental studies in animal models of atherosclerosis are required to unravel DC function, life cycle, activation, and trafficking in atherosclerosis. As DCs recognize atherosclerosis-related antigens and are renowned as potent immune-regulatory cells, they may possibly be manipulated and deployed in the future in order to delay the atherosclerotic disease process in humans. 

##  Authors' Contribution

E. A. Van Vré and I. V. Brussel contributed equally to the paper.

## Figures and Tables

**Table 1:** Immunohistochemical markers to identify DCs in human plaques.

Marker	DC type	References	Pitfalls	References
Fascin (p55)	Immature/mature DCs; DC specific in early plaque stages	[7, 59, 62, 65, 66, 68, 69, 73–77]	Capillary ECs, migrating vascular cells in plaque shoulders and advanced plaques	[69, 77]
S100 (S100B and weakly S100A1)	Immature/mature DCs; DC specific in normal intima and all plaque stages	[7, 7, 56, 59, 61, 65, 68, 69, 77–79]	Nerve bundles and twigs in adventitia	[7, 56, 59, 69]
Langerin	Selectively expressed on the surface and in Birbeck granules of Langerhans cells	[60, 69]	Very few cells	[60, 69]
CD1a	Mature DCs	[68, 74, 78, 80]	CD14^+^, CD68^+^ foam cells	[69, 81]
CD83	Mature DCs	[65–67]	Aspecific staining due to signal amplification? Activated T cells and monocytes?	[63, 69]
DC-SIGN (CD209)	Immature/mature DCs	[63, 64]	Macrophages	[63]
DC-LAMP (CD208)	Mature DCs	[65]		
BDCA-1 (CD1c)	mDC	[26, 68, 69]	B cells	[23]
BDCA-2 (CD303)	pDC precursor	[26, 68, 69]		[23, 38]
CD11c	mDCs	[71–73]	CD14^+^ monocytes and CD68^+^ macrophages	[69]
CD123	pDCs	[70–73]	ECs, microvessels in advanced plaques, and plaque shoulders	[69]

DC: dendritic cells, EC: endothelial cell, pDC: plasmacytoid DC, mDC: myeloid DC.
